# Susceptibility of *Duponchelia fovealis* Zeller (Lepidoptera: Crambidae) to Soil-Borne Entomopathogenic Fungi

**DOI:** 10.3390/insects9020070

**Published:** 2018-06-19

**Authors:** Rafaela F. Amatuzzi, Carolina G. Poitevin, Alex S. Poltronieri, Maria A. C. Zawadneak, Ida C. Pimentel

**Affiliations:** 1 Departamento de Patologia Básica, Universidade Federal do Paraná, Curitiba 81531-980, Paraná, Brazil; carol.poitevin@gmail.com (C.G.P.); mazawa@ufpr.br (M.A.C.Z.); ida@ufpr.br (I.C.P.); 2 Departamento de Fitotecnia, Universidade Federal de Santa Catarina, Florianópolis 88034-000, Santa Catarina, Brazil; alex.poltronieri@yahoo.com.br

**Keywords:** *Duponchelia fovealis*, strawberry, Crambidae, *Beauveria bassiana*, *Isaria javanica*, biological control, entomopathogenic isolates, IPM

## Abstract

*Duponchelia fovealis* (Lepidoptera: Crambidae) is an invasive species that has had a large impact on strawberry crops in Brazil. Pesticides have had limited effectiveness and the use of biological control agents to improve its management is the most appropriate approach. The aim of this study was to evaluate the pathogenicity and virulence of entomopathogenic fungi—isolated from soil—against *Duponchelia fovealis* larvae under laboratory and greenhouse conditions. Pathogenicity screenings were performed for twenty isolates from *Beauveria bassiana*, *Beauveria caledonica*, *Isaria javanica*, *Metarhizium anisopliae*, and *Lecanicillium* sp. against third instar larvae of *D. fovealis* at the concentration of 10^9^ conidia·mL^−1^. Lethal concentration (LC_50_) and lethal time (LT_50_) were determined for the most pathogenic isolates and for one commercial mycoinsecticide. Mortality rates varied from 10 to 89%. The isolates *B. bassiana* Bea1, Bea110, Bea111 and *I. javanica* Isa340 were the most pathogenic. The most virulent isolates were *B. bassiana* Bea111 and *I. javanica* Isa340 with LC_50_ values of 2.33 × 10^6^ and 9.69 × 10^5^ conidia·mL^−1^, respectively. Under greenhouse conditions, the efficacy of LC_50_ of the isolates *I. javanica* Isa340 and *B. bassiana* Bea111 were 45% and 52%, respectively. Our results indicate that these isolates are strong candidates for application in the control of *D. fovealis*. This study is the first evaluation of soil-borne entomopathogenic fungi against *D. fovealis*.

## 1. Introduction

*Duponchelia fovealis* Zeller (Lepidoptera: Crambidae) is a native species from the marshlands of southern Europe and has become one of the most destructive pests of greenhouse and strawberry crops in several countries, including Portugal [1], Italy [2], Turkey [3] and Brazil [4]. The larvae cause economic damage by feeding on the leaves, flowers, and crown, which results in wilting, collapse, and death of the plants and consequently reducing crop quality and yield [4]. Due to the need for daily picking of strawberry fruit and with the increasing awareness of problems associated with pesticide use, development of alternative control strategies have become the main goal in the integrated pest management (IPM) of pests such as *D. fovealis* [5,6,7]. Biological control is one of the most promising options [4,6,8], although there have been few studies on its application against *D. fovealis*. Entomopathogenic fungi (EPF) have attributes that make them strong candidates for use against this pest [9]. They are widespread in terrestrial environments, especially in the soil, which is considered a reservoir [10], and are important natural regulators of insect populations through infection by contact, consequently increasing the potential for epizootics and mortality rates in the pest population [11]. Fungi of the genera *Beauveria*, *Metarhizium*, and *Isaria* are widely known as biological control agents [12,13] and they are safe for farmers, consumers, and the environment [14,15,16]. Thus, preventive methods through integrated pest management can be used as an alternative to the chemical control of *D. fovealis*.

Traits such as virulence and pathogenicity are considered important properties of entomopathogens used in pest control [17]. Although entomopathogenic fungi have been widely used against a number of insect pests, no data are available on the efficacy of fungi against *D. fovealis*. This study aims to contribute to the implementation of an improved IPM program for this pest, with the first report of tested EPF isolated from native forest and crop systems soils against *D. fovealis* larvae under laboratory and greenhouse conditions.

## 2. Materials and Methods

### 2.1. Fungal Isolates and Insects

EPFs were isolated from soil samples collected between January and June 2015 from different geographical sites in the state of Parana, Brazil (Table 1). The isolates are deposited at the Paraná State Microbiological Collection Network—TAXONline (CMRP), Department of Basic Pathology, Federal University of Paraná, Curitiba, Brazil. Tests were conducted on third instar larvae of *D. fovealis* obtained from a stock colony reared on an artificial diet and maintained under controlled conditions of 25 ± 2 °C, 70 ± 10% relative humidity (RH), and a 14-h photophase [18].

### 2.2. Production of Fungal Conidial Suspensions

Each isolate (Table 1) was cultured on Sabouraud dextrose agar (SDA) medium and incubated at 25 °C in complete darkness for 15 days until sporulation was abundant. Conidia from each isolate were harvested under sterile flow conditions using a sterile steel spatula to scrape them into 30 mL glass tubes with 20 mL of 0.3% (*v*/*v*) Tween^®^ 80 solution. Conidial suspensions of each isolate were vortexed for 15 min at 120 rpm and then filtered, using sterile gauze (10 × 15 cm), into new 30 mL glass tubes. Suspensions were prepared independently for each assay and concentrations were adjusted by serial dilution to achieve a comparable number of conidia per milliliter in an aqueous 0.3% Tween^®^ 80 solution. Spore suspensions were used immediately after preparation and concentrations were adjusted using a hemocytometer. Spore viability was determined by direct counting [19,20]. Viability was then determined using a microscope (400× magnification) by visualization of 200 spores; spores that presented germinative tube growth were considered as viable. This procedure was adopted for all isolates and viability ranged from 90 to 95%.

### 2.3. Pathogenicity Bioassay

Strawberry leaves were disinfected superficially [21] and their petioles were wrapped in cotton and placed in a 20 mL glass tube with autoclaved sterile distilled water and then positioned in acrylic boxes (11 × 11 × 3.5 cm). The experimental design was completely randomized. Each treatment was conducted with five replications. Twenty-four third instar *D. fovealis* larvae were transferred to each box, so that each box was considered a replication, for a total of 120 larvae tested per treatment. A Sagyma SW776 airbrush (10 lb pol^−1^) was used to spray 1 mL of a conidial suspension at a concentration of 10^9^ conidia·mL^−1^ and Tween^®^ 80 at 0.3% over the leaves and larvae. The control treatment received 1 mL of sterile distilled water with 0.3% (*v*/*v*) Tween^®^ 80. After spraying, boxes were kept under controlled conditions (25 ± 2 °C, 70 ± 10% RH, and 14-h photophase). Mortality was assessed after 7 days. Moribund individuals or those that did not respond to touch with a paintbrush were considered dead. These specimens were transferred to Petri dishes with moistened filter paper until fungal extrusion to check for postmortem sporulation. The experiment was carried out for 5 weeks.

### 2.4. Virulence Bioassay and Determination of LC_50_

Fungal strains that caused high mortality rates of third instar larvae *D. fovealis* were selected to determine lethal concentration (LC_50_) and lethal time (LT_50_). Five concentrations were tested: 1 × 10^4^; 1 × 10^5^; 1 × 10^6^; 1 × 10^7^, 1 × 10^8^ conidia·mL^−1^. For each concentration, 120 larvae were used, divided into five replicates. The control treatment consisted of sterile distilled water added 0.3% (*v*/*v*) Tween^®^ 80. A commercially available biopesticide (Bovemax^®^) was used for comparison, containing *B. bassiana* CG716 strain as the active ingredient. Spraying was conducted as described for the pathogenicity bioassay and the samples were monitored daily for 7 days. Mortality assessments were also conducted as described above.

### 2.5. Greenhouse Assay

The fungi that presented the lowest LC_50_ (between 1 × 10^5^ and 1 × 10^6^ conidia·mL^−1^) in the laboratory bioassays were selected for the greenhouse assay. Healthy 3-month-old strawberry seedlings of the ‘Albion’ variety were transferred into 500 mL containers with the Plantmax^®^ potting mix. The plants were kept under natural light and watered twice a day. During the assay, minimum, maximum, and 24-h mean temperatures in the greenhouse were recorded daily with a digital thermometer (Incoterme). The experimental design was completely randomized. Each treatment was tested on 20 seedlings, each considered a replicate. The plant leaves were sprayed with 33 mL of fungal suspension of the selected isolates using a CO_2_ precision sprayer (Teejet XR11008VS, Spraying Systems Company, Wheaton, IL, USA). This volume corresponds to 200 L ha^−1^ with a density of 60,000 plants, as recommended by manufacturers of most commercial biopesticides. The control was sprayed with sterile distilled water containing Tween^®^ 80 at 0.3% (*v*/*v*). Spraying was carried out from the bottom upwards to ensure coverage of all surfaces. Each plant was then infested with 10 third instar larvae of *D. fovealis* and isolated in cages covered by voile fabric. To prevent insects from escaping through the soil, they were isolated by a transparent laminated plastic disc (30 cm) covering the entire surface of the pot with a small central opening for the plant stem. Mortality was assessed after 7 days.

### 2.6. Statistical Analyses

In the pathogenicity test, mean mortality rates (%) were used to calculate fungal efficiency according to Abbott’s equation [22] and the treatments were compared using Tukey’s test (*p* < 0.05). Estimated LC_50_ and LT_50_ were calculated by Probit analysis using PoloPlus software [23]. For the greenhouse assay, the mean number of insects per strawberry plant was analyzed with ANOVA and Tukey’s test (*p* < 0.05). When differences were significant compared to the control group, efficacy was calculated according to Henderson and Tilton’s formula [24].

## 3. Results

### 3.1. Pathogenicity Bioassay

**Figure 1:**
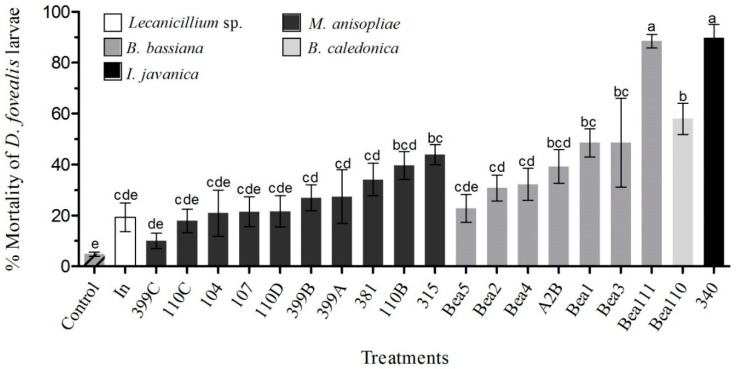
Adjusted mortality rate (mean ± SE) of third instar larvae of *D. fovealis* 7 days after inoculation with conidial suspensions (10^9^ conidia·mL^−1^) of entomopathogenic fungi (EPF) isolates from different soils. Bars with the same letter are not significantly different according to the Tukey test (*p* < 0.01).

Mortality of *D. fovealis* larvae varied between 10% and 89% (Figure 1). Of the 20 fungi tested (Table 1), thirteen isolates caused significant mortality compared to the control group (4.76%) (F_20,84_ = 10.43, *p* < 0.01). The treatments with *B. bassiana* (Bea1, Bea3, Bea111), *B. caledonica* (Bea110), and *I. javanica* (Isa340) induced the highest mortality rates, which varied between 48% and 89%. For the *B. bassiana* isolates Bea2, Bea4, and A2B and *M. anisopliae* 381, 110B, 399A, 399B, and 315, the mortality ranged between 27% and 45%. *M. anisopliae* isolates 399C, 110C, 110D, 104, 107, *Lecanicillium* sp. (In), and *B. bassiana* Bea5 were not significantly pathogenic with mortality rates below 22%. Fungal extrusion was observed in all larvae with various degrees of mycelial growth among the treatments at the end of 7 days of incubation.

### 3.2. Virulence Bioassay

**Figure 2:**
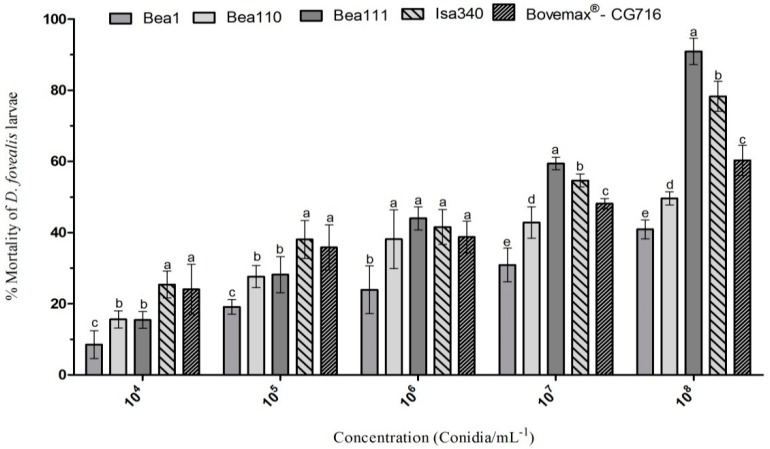
Adjusted mortality of third instar larvae of *Duponchelia fovealis* after inoculation with varying concentrations of conidia of five strains of entomopathogenic fungi: *B. bassiana* Bea1 and Bea111, *B. caledonica* Bea110, *I. javanica* Isa340, and Bovemax CG716. Error bars represent confidence intervals at 95%. Tukey test was performed separately for each concentration and bars with the same letter are not significantly different (*p* < 0.001).

The proportion of insects infected increased with the conidial concentrations tested in *D. fovealis* larvae. There was a significant mortality of *D. fovealis* larvae among isolates for each concentration, 10^4^ (F_4,24_ = 20.77, *p* = 0.001), 10^5^ (F_4,24_ = 20.11, *p* = 0.001), 10^6^ (F_4,24_ = 5.40, *p* < 0.004), 10^7^ (F_4,24_ = 96.39, *p* < 0.001), and 10^8^ (F_4,24_ = 269.96, *p* < 0.001), although the increased concentrations did not produce the same effect for all fungal isolates (Figure 2). Significant differences in the mortality between *B. bassiana* Bea111 and *I. javanica* Isa340 isolates were found in 10^7^ and 10^8^ conidia·mL^−1^ concentrations. The *Beauveria bassiana* isolate Bea1 caused the lowest infection rates in larvae compared with the other isolates at all conidial concentrations and Bovemax^®^ obtained mortality similar to *I. javanica* isolate Isa340 at all concentrations except 10^7^ and 10^8^ conidia·mL^−1^.

Isolates Isa340 (*I. javanica*) and Bea111 (*B. bassiana*) presented the lowest effective concentrations (Table 2) with lethal concentrations and average times of 9.69 × 10^5^ conidia·mL^−1^ in 6.4 days and 2.33 × 10^6^ conidia·mL^−1^ in 7.2 days, respectively. The LC_50_ for the commercial isolate Bovemax^®^ (*B. bassiana*) was 1.41 × 10^7^ conidia·mL^−1^ with a similar LT_50_ (7.5 days). The results for the isolates Bea110 (*B. caledonica*) and Bea1 (*B. bassiana*) were less promising with higher lethal concentrations.

### 3.3. Greenhouse Assay

Isolate Bea111 (*B. bassiana*) and Isa340 (*I. javanica*) significantly reduced insect populations. Larval mortality differed significantly between treatments and the control group (F_2,48_ = 12.04, *p* < 0.05). The first isolate reduced larval infestation by 52% compared to the control group while the second isolate caused a 45% reduction in the initial population. Fungal extrusion occurred on average after 5 days (Table 3). The mean temperature during the assays was 20.5 °C (18.3–24.0 °C) with a minimum of 16.2 °C (16.0–18.0 °C) and a maximum of 31.2 °C (29.7–33.0 °C).

## 4. Discussion

Several species of entomopathogenic fungi were evaluated for their potential as pest control agents, with promising results of different pests for several crops that have already been extensively studied [25,26,27,28]. However, studies on the biological control of *D. fovealis* in strawberry crops are new. Our results have indicated that among the 20 surveyed isolates that are pathogenic for *D. fovealis*, the mortality rates varied between 10% and 89%. Such variation in mortality rates is expected when several isolates obtained from the environment are tested. A similar result was observed in a previous in vitro study using endophytic fungi against *D. fovealis* larvae, with mortality rates varying between 30% and 80% (10^9^ conidia·mL^−1^) [9], reinforcing the argument that the use of entomopathogenic fungi may be effective against this pest. Another study that evaluated *B. bassiana* and *M. anisopliae* against larvae of *Plutella xylostella* L. (Lepidoptera: Plutellidae) also obtained mortality rates varying between 20% and 94% (10^8^ conidia·mL^−1^) [29].

Although *Beauveria caledonica* (Bea110) produced over 57% mortality in the pathogenicity tests, this isolate did not present high virulence on *D. fovealis*, although it had a shorter LT_50_ of approximately 5 days. This might be due to the ecology of this species since *B. caledonica* presents a more restricted host relationship with coleopterans. *Beauveria caledonica* is naturally found in soil and has already been recorded as being highly pathogenic to weevils with mortality rates between 70% and 90% (10^7^ conidia·mL^−1^) and an average LT_50_ between 6 and 8 days for *Hylobius abietis* [30].

In our study, *Metarhizium anisopliae* isolates induced mortality rates between 10% and 43%, unlike studies on biological control with isolates of *M. anisopliae*, which reported high mortality rates [31,32]. The low performance of *M. anisopliae* may be due to the selectivity of certain strains to particular hosts as targets [33]. Other studies using other Lepidoptera species obtained similar results [29].

The isolate Bea111 presented a low value of LC_50_ compared with the commercial strain Bovemax^®^. The LC_50_ found in the present study was similar to other virulence studies using *B. bassiana* where an average LC_50_ between 10^4^ and 10^6^ conidia·mL^−1^ was found [25,34].

*Isaria javanica* Isa340 presented the lowest LC_50_ value among all evaluated isolates, reinforcing the great potential of this entomopathogenic genus for pest control. The genus *Isaria* is considered highly virulent for hosts of several orders of insects [35]. Studies on *Isaria fumosorosea* against larvae of *Plutella xylostella* (Lepidoptera: Plutellidae) [36] and *Isaria poprawskii* against nymphs of *Homalodisca vitripennis* (Hemiptera: Cicadellidae) [37] showed LC_50_ close to the values observed in our study.

Most entomopathogenic fungi perform notably well under laboratory conditions and high levels of mortality can be observed for most insect targets under such controlled conditions. An unfortunate consequence of such reports is that this establishes unrealistic expectations regarding the performance of these agents in the field. Variations in temperature, humidity, and exposure to UV-irradiation are amongst some of the important abiotic factors that can significantly impact the efficacy of entomopathogenic fungi in field applications [15]. Our results under greenhouse conditions also confirmed the performance of isolates Bea111 and Isa340, which induced mortality rates between 45% and 52%. In addition, the behavior presented by larvae of *D. fovealis*, which lodges itself at the base of the strawberry plants [4], along with the plant structure itself, may construct a favorable habitat that contributes to the effectiveness of the pathogens [38]. Several species of entomopathogenic fungi have been evaluated as pest control agents under laboratory and greenhouse conditions or directly in the field, with promising results in different pests of several crops that were already widely studied [25,26,27,28,39]. However, this is the first report on evaluations of soil-borne entomopathogens against *D. fovealis* in these conditions.

## 5. Conclusions

In conclusion, our results indicate that isolates *Beauveria bassiana* Bea111 and *Isaria javanica* Isa340 are strong candidates for further evaluation in the control of *Duponchelia fovealis* and present good potential for the development of products as mycoinsecticides. Our results represent the first report of entomopathogenic fungi isolated from soil infecting *D. fovealis*, and the basis for future studies to design a biological control program in strawberry crops.

## Figures and Tables

**Table 1:** Entomopathogenic fungi isolates tested against *Duponchelia fovealis* and the GenBank accession numbers for their respective ITS sequences.

Species	Isolate	*GenBank*	Soil Source	Coordinates and Sites
*Beauveria bassiana*	Bea1	KY471,648	Conventional corn (*Zea mays*) crop systems	25°53′190″ S, 49°43′195″ W Araucária
	Bea2	KY471,649		
	Bea3	KY471,650		
	Bea4	KY471,651		
	Bea5	KY471,652		
*Beauveria caledonica*	Bea110	KY471,655	Conventional strawberry (*Fragaria* × *ananassa*) crop systems	25°53′376″ S, 49°44′654″ W Araucária
*Beauveria bassiana*	Bea111	KY471,653	Conventional corn (*Zea mays*) crop systems	
	A2B	KY471,654		
*Lecanicillium* sp.	In1	KY471,666	Organic strawberry (*Fragaria* × *ananassa*) crop systems	25°74′010″ S, 49°89′425″ W Lapa
*Metarhizium anisopliae*	104	KY471,656	Native forest	25°34′154″ S, 48°90′083″ W Morretes
	107	KY471,657		
*Isaria javanica*	Isa340	KY488,507		
*Metarhizium anisopliae*	110B	KY471,658	Banana trees (*Musa* spp.) in native forest	25°38′776″ S, 48°86′026″ W Morretes
	110C	KY471,659		
	110D	KY471,660		
	315	KY471,661		
	381	KY471,662		
	399A	KY471,663		
	399B	KY471,664		
	399C	KY471,665		

**Table 2:** Probit regression analysis of the mortality and log-concentration for different entomopathogenic fungi (EPF) isolates against third instar larvae of *D. fovealis*.

Treatment	Bea1	Bea110	Bea111	Isa340	CG716
Number of larvae	601	557	609	645	432
LC_50_	9.5 × 10^8^	3.85 × 10^8^	2.33 × 10^6^	9.69 × 10^5^	1.41 × 10^7^
Slope (SE)	0.24 ± 0.06	0.25 ± 0.05	0.51 ± 0.04	0.37 ± 0.03	0.21 ± 0.05
95% FL	3.7 × 10^8^–5.09 × 10^9^	5.13 × 10^7^–5.6 × 10^9^	8.88 × 10^5^–5.34 × 10^6^	5.79 × 10^5^–3.12 × 10^6^	4.48 × 10^6^–8.38 × 10^7^
χ^2^ (df = 4)	2.56	1.27	3.65	2.77	0.29
LT_50_ (Days)	7.1	5.3	7.2	6.4	7.5
Slope (SE) 95% FL	2.10 ± 0.27 6.0 to 9.0	2.16 ± 0.25 4.6 to 6.2	3.13 ± 0.38 6.4 to 8.6	2.81 ± 0.30 5.4 to 8.3	2.75 ± 0.39 6.4 to 9.6

**Table 3:** Efficacy of *Beauveria bassiana* (Bea111) and *Isaria javanica* (Isa340) after 7 days of treatment on third instar larvae of *Duponchelia fovealis* in strawberry plants in greenhouse conditions. Values followed by the same letters are not significantly different according to the Tukey test (*p* < 0.05).

Treatment	N° Initial Larvae	% Efficacy (SE)
Bea111 (2 × 10^6^) *	197	52.68 (6.23) a
Isa340 (9 × 10^5^) *	196	45.83 (7.79) a
Control	199	17.41 (3.11) b

* Concentration (conidia·mL^−1^).
